# Current Devices in Mitral Valve Replacement and Their Potential Complications

**DOI:** 10.3389/fcvm.2020.531843

**Published:** 2020-11-27

**Authors:** Livia Gheorghe, Jorn Brouwer, Dee Dee Wang, Nina Wunderlich, Bushra Rana, Benno Rensing, Frank Eefting, Leo Timmers, Martin Swaans

**Affiliations:** ^1^St. Antonius Hospital, Nieuwegein, Netherlands; ^2^Henry Ford Hospital, Detroit, MI, United States; ^3^Cardiovascular Center Darmstadt, Darmstadt, Germany; ^4^Royal Papworth Hospital NHS Foundation Trust, Cambridge, United Kingdom

**Keywords:** mitral valve, mitral replacement, complication, minimal invasive approach, LVOT obstruction

## Abstract

Mitral regurgitation is one of the most prevalent valvulopathies worldwide, and its surgical treatment is not feasible in all cases. The elderly and frail with several comorbidities and left ventricular dysfunction are often managed conservatively. Percutaneous treatment (repair or replacement) of the mitral valve has emerged as a potential option for those patients who are at a high risk for surgery. Mitral valve repair with the Mitraclip device proved both increased safety and mortality reduction in patients with severe mitral regurgitation. On the other hand, in the last decade, percutaneous mitral replacement opened new frontiers in the field of cardiac structural interventions. There are few mitral devices; some are in the early phase of development and some are waiting for CE mark of approval. The evolution of these devices was more complicated compared to the aortic technology due to the native mitral valve's complexity and access. This review aims to provide an overview of the current devices, their specific features, and their potential complications.

## Introduction

Opposite to transcatheter aortic valve replacement (TAVR), transcatheter mitral valve replacement (TMVR) is a much more complex procedure due to the mitral valve's anatomy and shape, lack of calcification, and its relationship with adjacent structures. An adequate pre-procedural study is mandatory and comprises of multimodality imaging to define mitral regurgitation, to evaluate a patient's eligibility according to anatomic characteristics, to plan the implantation access, and to identify possible issues during TMVR. There are few serious challenges such as mitral valve position, valve sealing, the proximity of the left ventricular outflow tract (LVOT), delivery system size, prosthesis anchoring, and valve thrombogenicity. Initial studies have shown encouraging results; nevertheless, the mortality at 1-year follow-up is high (1–4). Although the present valves have different mechanisms of anchoring, the principal access is still transapical, which may be deleterious due to the negative effects of thoracotomy in an elderly population with a higher degree of myocardial injury, especially in patients with reduced LVEF pre-procedurally.

The present review aims to describe principal transcatheter mitral valves, focusing on their mechanism, anchoring design, and the potential complications that can occur during TMVR.

## What Kind of Mitral Pathology Can We Treat Percutaneously?

Mainly, the most frequent pathology on the mitral valve is mitral regurgitation (MR), which may be either degenerative or functional (5). A high number of patients with severe MR do not receive surgical treatment due to the high risk and comorbidities (5, 6).

On the other hand, mitral annular calcification (MAC) is a degenerative process, affecting the fibrous base of the mitral valve, and its prevalence reaches 15% (7).

MAC may be associated with regurgitation or stenosis. Surgical treatment of this particular entity is complex due to the risk of potential complications such as intractable hemorrhage, ventricular rupture, or atrioventricular disruption, even for experienced cardiac surgeons (8).

Moreover, up to 25% of mitral bioprostheses present degeneration at 15-year follow-up (9), and 15% of mitral repairs have moderate–severe MR at 20-year follow-up (10). Reoperation has an additional surgical risk, especially in elderly patients.

In these scenarios (mitral regurgitation/stenosis in high-risk patients, MAC, and previous mitral replacement of repair), TMVR may play an important role, but extensive knowledge of the mitral valve anatomy is imperative and a rigorous screening should be done to evaluate the procedure feasibility.

### Complex Anatomy, Complex Valve Design

The mitral valve apparatus is mainly composed of the mitral annulus, two leaflets, left atrium, left ventricle (LV), papillary muscles, and tendinous chords. Any disturbance of these components may determine mitral valve dysfunction.

The mitral annulus is rather a concept than an anatomical structure, and its characteristics are determinant for mitral valve replacement. The D shape with 3D geometry and size change with each cardiac cycle are just a few items that should be taken into consideration during transcatheter heart valve (THV) development (11). The lack of calcification makes the anchoring of the new valve difficult. There are several valves with a distinct anchoring mechanism to ensure good position and sealing. The majority present a system which anchor the valve at the level of the mitral annulus [grasping the leaflets (12) or clamping the annulus (13)], and others such as the Tendyne valve offer an anchoring system connected to the LV apex through a tether (14).

### Pre-procedural Assessment for the TMVR

Identifying suitable candidates for TMVR therapy has been a challenge for all devices. There are multiple exclusion criteria which can be clinical, anatomical, and/or device specific (Table 1). The most frequent exclusion criteria are anatomic, and studies such as coronary angiography, transthoracic and transesophageal echocardiography (TTE/TEE), and cardiac computer tomography (CT) are mandatory for patient selection. Moreover, imaging is fundamental for both diagnostic and procedure guiding. Echocardiography is the main tool for mitral valve evaluation. TTE gives information regarding the thickness of the interventricular septum, which is essential to determine if there is a risk for LVOT obstruction. Coronary angiography permits evaluation of the septal branches in case alcohol ablation is required.

**Table 1:** Published exclusion criteria for current transcatheter mitral valve replacement.

**Devices enrolling studies**	**LVEDD (mm)**	**LVEF (%)**	**Severe mitral leaflet calcification**	**Unsuitable chordal anatomy**	**High risk of LVOT obstruction**	**Unfavorable mitral valve anatomy**	**Severe tricuspid regurgitation**	**Severe pulmonary hypertension**	**Others**
Tendyne (NCT02321514)	>70 mm	<30%	X		X	X	X	X	NA
Intrepid (NCT02322840)	NA	<20%	X	NA*	NA	NA*	NA	X	Severe renal insufficiency, and prior mitral valve surgery or intervention
TIARA (NCT03039855)	NA	<20%	X	N/A	Area <2.0 cm^2^ at end-systole	NA	NA	X	Severe right ventricle dysfunction
CardiaQ (NCT02722551)	NA	NA	X	X	NA	X	NA	NA	Previous aortic valve replacement
Sapien M3 (NCT03230747)	>70 mm	<30	X	X	X	X	NA	NA	NA
Caisson (NCT02768402)	>70 mm	NA	X	X	X	X	NA	NA	Severe right ventricle dysfunction
Highlife *NCT02974881*	>70 mm	<30	X	X	X	X	NA	NA	NA
Fortis	NA	NA	X	X	X	X	NA	NA	NA
CardioValve NCT03813524	>75	NA	X	X	X	X	X	X	NA
Evoque	NA	<30%	X	X	Area <1.5 cm^2^ at end-systole	X	X	X	NA

*NA, not available; X- exclusion criteria*.

* *Native mitral valve geometry and size compatible with the Intrepid™ TMVR*.

CT imaging is essential during pre-procedural planning for TMVR because it provides almost all the information needed to plan the procedure.

Specific measurements should be done for each component of the mitral valve apparatus.

Intercommisural, septal-to-lateral, trigone-to-trigone distance, and 3D perimeter are useful to size the adequate valve. Some of the devices rely on intercommissural distance such as the Tiara® valve (Neovasc Inc; Richmond, BC) (11) or on maximum diameter such as the CardiaQ® valve (Edwards Lifesciences; Irvine, CA) (15). The intrepid valve (Medtronic, Minneapolis, Minnesota) size is obtained by oversizing the mitral annular perimeter, inter-commissural diameter, and septal-lateral diameter (16) by 10 to 30%. The extent and the location of mitral annular calcifications should also be reported. Asymmetrical annular calcification may interfere during valve implantation, with a higher risk of embolization if it is present along the device-grasping zone. Protruding calcification of the anterior leaflet can be displaced into the LVOT, causing LVOT obstruction (17). The length of the leaflets, especially the anterior one, is important. It may be pushed by the device and obstruct LVOT even in the absence of calcification. Other distances are also necessary: the distance between the papillary muscle heads, the projected distance to the mitral annulus plane, and the distance to the ventricular wall. Since most valves start to expand into the left atrium, it is mandatory to know if there is enough space to deploy the device (left atrium height, short and long axis, left atrial appendage ostium to mitral annulus distance). The LVOT obstruction is one of the most fearsome complications during TMVR; therefore, the Neo-LVOT cross-sectional area and the aorto-mitral angle should be assessed. A normal angle is about 120° in peak systole, and if it is narrower, it may predispose to LVOT obstruction post-TMVR.

A thorax CT scan can provide valuable data regarding the ideal intercostal space for the trans-apical approach and the angulation for coaxial deployment. Finally, an abdominopelvic CT scan provides information on ileo-femoral vein access in case of a transfemoral approach.

### Transcatheter Valve Devices for Mitral Replacement

**Figure 1:**
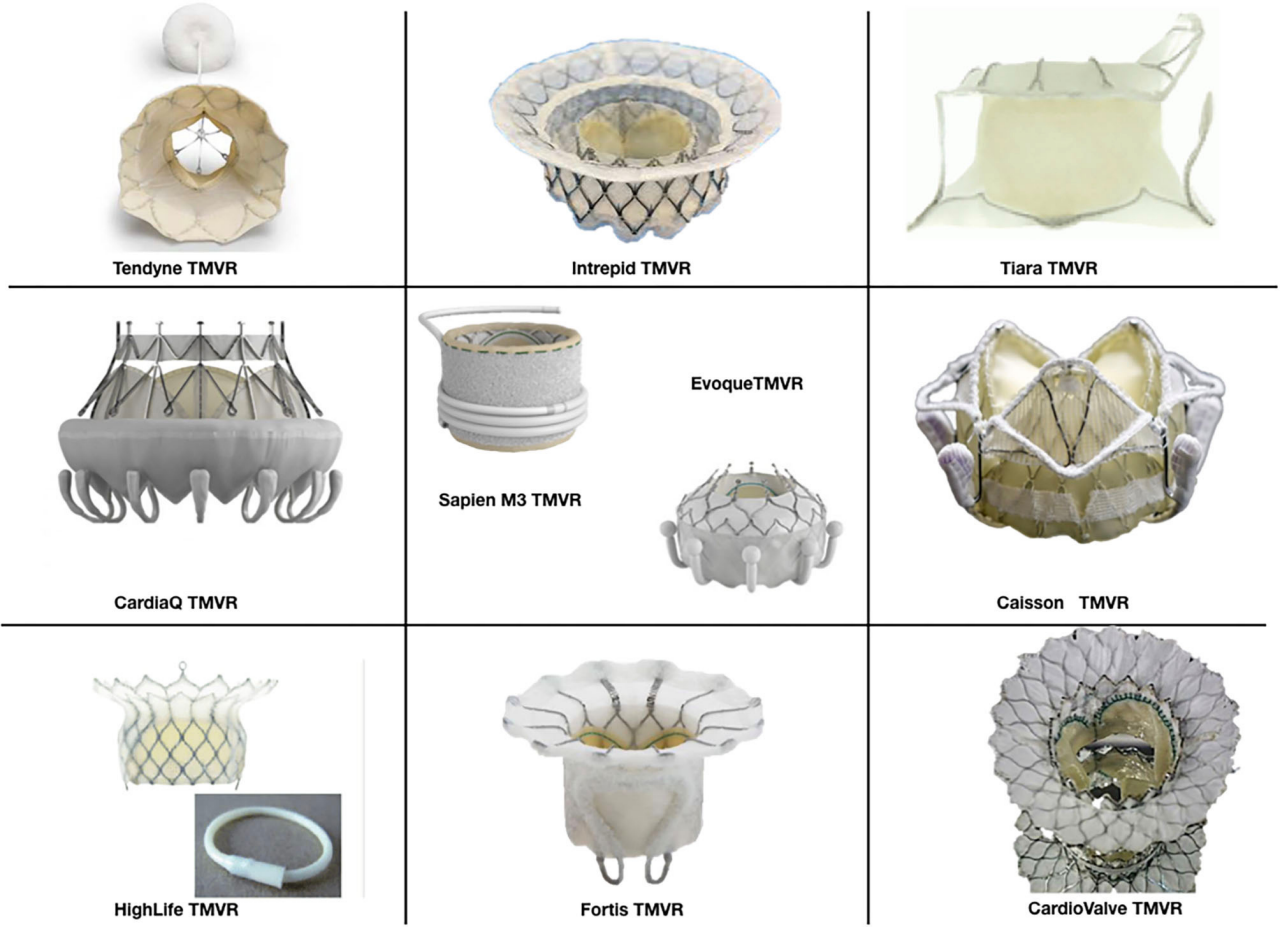
Percutaneous mitral valves devices.

There are at least 16 devices developed for percutaneous mitral valve replacement. Most of these devices are in the early phase of development and do not have Food and Drug Administration (FDA) approval or the CE Mark (Figure 1). They can only be used in studies or for compassionate use cases (Table 2). The results of the feasibility studies (1, 14) of the Tendyne TMVR were positive and led to CE mark approval. Although mitral pathology is very prevalent, the inclusion rate is still low due to the multiple exclusion criteria (Table 1).

**Table 2:** Transcatheter mitral valve devices.

**Device**	**Manufacture**	**Access size sheath**	**Anchoring mechanism**	**Valve size**	**Effective orifice area**	**Valve position**	**Recapture**	**Shape**	**Frame**	**Leaflets**
Tendyne	Abbott	TA 34 Fr.	Apical tether	Outer (sealing) frame ranges 30–43 mm in the SL dimension and 34–50 in the IC dimension	3.2 cm^2^	Intra-annular	Fully recapturable system after complete deployment	D-shaped (outer stent) Circular (inner frame)	Nitinol, double frame; Self-expandable	Porcine pericardium, trileaflet
Intrepid	Medtronic	TA 33Fr.	Radial force and sub-annular cleats	Inner stent−27 mm (Outer stent−43, 46, and 50 mm)	2.4 cm^2^	Intra-annular	No	Circular	Double stent, self-expanding, nitinol	Bovine pericardium, trileaflet
TIARA	Neovasc	TA 32, 36 Fr.	3 ventricular anchoring tabs (onto the fibrous trigone and posterior shelf of the annulus)	35 and 40 mm	6.5–12 cm^2^	Intra-annular	No	D-shaped	Self-expanding, nitinol	Bovine pericardium, trileaflet
CardiaQ	Edwards Lifesciences	TA/TF 33 Fr.	Mitral annulus capture with native leaflet engagement	30 mm	NA	Supra-annular	No	Circular	Self-expanding, nitinol	Bovine pericardium, trileaflet
Sapiens M3	Edwards Lifesciences	TF/TA 20 Fr.	Nitinol dock system	29	NA	Intra-annular	No	Circular	Balloon-expandable, cobalt-chromium frame	Bovine pericardium, trileaflet
Caisson	LivaNova	TF 31 Fr.	4 sub-annular anchoring feet 3 atrial holding features	36A 42A 42B	NA	Supra-annular	Recapturable/ retrievable	D-shaped	2 components (anchor and valve); Nitinol, self-expandable.	Porcine Pericardium, trileaflet
HighLife	HighLife SAS	TA39 Fr. (TF artery for loop placement)	External anchor; valve in sub-annular mitral ring	31 mm	NA	Intra-annular	No	Circular	2 components (ring and valve); Nitinol, self-expandable	Bovine pericardium, trileaflet
Fortis	Edwards Lifesciences	TA 42 Fr.	2 opposing paddles	29 mm	NA	Intra-annular	No	Circular	Cloth-covered, self-expanding, nitinol	Bovine pericardium, trileaflet
CardioValve	CardioValve	TF 28-Fr.	24 focal “sandwiching” points	3 sizes (range 40–40 mm)	NA	Intra-annular	No	Circular	Dual nitinol frame	Bovine pericardium, trileaflet
Evoque	Edwards Lifesciences	TF 28-Fr.	External anchor	2 sizes (44 and 48 mm)	NA	Intra-annular	No	Circular	Self-expanding, nitinol with fabric skirt to minimize paravalvular leak	Bovine pericardium,trileaflet

*Fr, French; TA, transapical; TF, transfemoral*.

There are four scenarios of mitral pathology treated with TMVR: (1) native non-calcified valves with severe mitral regurgitation, (2) native calcified valve (valve in MAC—Vi-MAC) with either mitral regurgitation or stenosis, (3) failed prosthetic ring and band (valve in ring, MViR), and (4) failed bioprosthesis (valve in valve—MViV). Specific devices were designed for the first scenario. For Vi-MAC, aortic THV devices and some of the mitral THV are currently used in compassionate use cases (18). Moreover, the aortic THV devices are the only ones used for both MViR and MViV.

### Devices for Native Non-calcified Mitral Valves

#### Tendyne Mitral Valve System

The Tendyne system (Abbott Structural, Santa Clara, California) is one of the specific THV devices designed for the mitral valve with the most significant worldwide experience. Nevertheless, so far, <1,000 valves have been implanted.

It is a unique valve-tether-pad design, with multiple valve sizes and profiles to address a range of pathoanatomy. The trileaflet porcine pericardial valve is mounted on a self-expanding nitinol double-frame stent and anchored to the left ventricle apex through a tether.

The inner stent is one size and circular to maintain an effective orifice area of >3.2 cm^2^, while the outer frame is D-shaped to conform with the shape of the mitral annulus (Table 2). A polyethylene terephthalate cuff aid at the atrial level provides valve sealing (the anterior extension above the plane of the annulus prevents paravalvular leak) and anchoring (preventing valve embolization into the ventricle when force is applied to the tether) (19). The valve is repositionable and fully retrievable, with no need for rapid pacing during deployment.

The procedure uses a transapical approach, of which the site and the trajectory are determined by pre-procedural CT and intraoperative TEE imaging (14). Once access is obtained, a balloon-tipped catheter is advanced into the left atrium to deliver a standard 0.035-in. guidewire. The delivery system and the valve are advanced and positioned above the mitral annulus, allowing partial device expansion. The valve orientation and expansion is checked with the TEE, ensuring that the outer frame fits into the mitral annulus and is aligned with the straight edge oriented anteriorly against the aorto-mitral continuity (11). The implantation technique is very important, and it was observed that a shorter Tendyne apical pad distance to the true apex was associated with better reverse remodeling after TMVR (20). The Early Feasibility Study of the Tendyne Mitral Valve System (NCT02321514) (1) showed encouraging results. The intervention was safe, without any mortality, and with a technical success rate of 96%. The mortality at 1-year follow-up was 27.6% (80% cardiovascular death), and it was similar to the interventional group of the MITRA FR study (21). Bleeding and re-hospitalization for heart failure were the most common complications even after 30 days. Prosthesis thrombosis and hemolyses due to paravalvular leakage were observed in 6 and 3% of cases, respectively. Moreover, 7% of a total of 100 patients needed pacemaker implantation. Nevertheless, at 1-year follow-up, 98.4% of patients had no mitral regurgitation, and 88.5% were in NYHA functional class II or I.

Finally, the SUMMIT trial (NCT03433274) is currently enrolling patients and is composed of three trial cohorts: randomized, non-randomized, and MAC. The subjects in the randomized cohort will be randomized in a 1:1 ratio to the trial device or the MitraClip system, and those in the non-randomized and MAC groups will receive the trial device.

#### Medtronic Intrepid TMVR

The Intrepid™ system is probably the second most used mitral THV. It is composed of an outer stent frame (also called the fixation frame), which has a flexible atrial portion, allowing conformability with the native mitral annulus, and a stiffer ventricular portion, which is wider than the native annulus. The inner stent frame houses a one-size 27-mm trileaflet bovine pericardium valve, ensuring an effective orifice area of >2.4 cm^2^ (16). The outer stent frame has three sizes (43, 46, and 50 mm). Additionally, it has cleats designed to engage the native mitral leaflets and a flexible atrial brim to facilitate echocardiography visualization. The principal advantages of this system are the unique anchoring system with a “champagne cork-like” effect produced by a radial force along the valve stent and the height of the valve (18 mm), which reduces the risk of LVOT obstruction (22). Currently, the valve can only be implanted through transapical access using a 35-Fr sheath (Table 2). The procedure is guided with TEE and fluoroscopy. First, a mini-left thoracotomy is performed, and a 7-Fr sheath is introduced into the left ventricle over a wire. Lately, it is exchanged for the device delivery catheter, which reaches the left atrium. The atrial brim is expanded using hydraulic delivery and then aligned with the mitral annulus, taking care to maintain the brim into the left atrium. The valve is deployed under rapid ventricular pacing. Once the valve is implanted, the delivery system is then withdrawn from the left ventricle, and the apical access site is closed. The first experience and the mid-term follow up results were recently published (22). The study included 50 patients with severe MR and at a very high risk for surgery (mean STS score: 6.4 ± 5.5). The successful rate implantation was 98%. During the follow-up (173 days; interquartile range, 54 to 342 days), 11 patients (22%) died (100% cardiovascular mortality). Bleeding was the most common complication and mainly related to the access site, with a need for re-intervention in five patients. No case of embolization or late paravalvular leak of hemolysis was described. The procedural survivors experienced improvements in symptom status and quality of life, and 73.8% had no mitral regurgitation at the time of the last follow-up (the rest presented trivial MR) (22). Apollo Trial (NCT03242642), which started in 2017, is a multi-center, global, prospective, randomized, interventional, and pre-market trial with two groups. In the randomized group, the patients may receive either the study device or conventional mitral valve surgery. Recently, the trial presented some changes, including an edge-to-edge repair group in the randomized cohort. The subjects in the single-arm group (ineligible for a surgical procedure) will receive the study device. Moreover, in this cohort, patients with MAC will be included in the TMVR MAC registry. Finally, at the end of 2019, the FDA approved an early feasibility study for the new Intrepid system using transfemoral approach.

#### Neovasc Tiara Mitral TMVR

The Tiara™ system (Neovasc Inc., Richmond, BC, Canada) is a new percutaneous transcatheter mitral trileaflet valve. It is mounted on a nitinol self-expanding platform presenting. The frame is D-shaped, with an atrial part composed of an asymmetric skirt, which provides anchoring and sealing characteristics. The principal mechanism of anchoring is provided by the three ventricular tabs, two anteriorly and one posteriorly (11). The posterior tab anchors onto the posterior shelf of the annulus, and the anterior tabs anchor onto the aortomitral fibrous trigone. The valve has two sizes, with a large effective orifice area (6.5–12 cm^2^) (12) (Table 2).

As the Tendyne and the Intrepid, the Tiara valve is implanted through transapical access (left mini-anterior thoracotomy) under TEE and fluoroscopic guidance. After the LV puncture, a 0.035-in. J wire is advanced across the mitral valve into the left atrium and exchanged for a 0.035-in. Amplatz Extra-Stiff™ wire. The TIARA TMVR delivery system is inserted across the MV into the left atrium, and subsequently, the atrial skirt of the TIARA system is unsheathed. At this moment, 3D TEE is fundamental to orientate the valve and ensure a perfect anatomical alignment of the D-shaped device with the geometry of the MV annulus. Finally, the ventricular portion and the anchoring tabs are released with further unsheathing of the system. Re-sheathing, repositioning, and retrieval can be safely performed before the release of the ventricular skirt. After the deployment, the delivery system is re-sheathed and removed from the LV apex. The latest published data regarding the TIARA valve included 73 patients (22 patients were compassionate use cases) (23). The procedural success rate was 93% (three cases of device malpositioning and two cases of valve migration) with 30-day mortality of 11.2%. Although one-third of the patients were compassionate use cases, there was no procedural mortality. Moreover, during follow-up, 88% of the patients were free of important mitral regurgitation (23).

#### CardiaQ-Edwards TMVR

The CardiAQ-Edwards transcatheter mitral valve is the first THV implanted *via* transfemoral approach back in 2012 (24). Later, the valve was also available for transapical access.

The principal structure is a self-expanding nitinol frame with a 30-mm diameter at the inflow and 40 mm at the annulus, covering the native mitral annular dimensions from 36 to 39.5 mm. The frame presents two sets of opposing anchors, which will be engaged at the level of the native annulus and leaflets to secure the valve. The bioprosthesis contains a trileaflet valve from bovine pericardial tissue. Additionally, the valve presents two skirts (at the level of the inflow and the outflow aspects of the frame) to reduce possible paravalvular leaks (Table 2). The device also contains an additional band at the level of the inflow aspect for bigger stability (25). The transfemoral and the transapical implantations follow almost the same steps: once access is obtained, the delivery system is advanced across the interatrial septum and apex, respectively. Later, the device crosses the mitral valve, and ventriculography is performed to find the proper mitral plane and correct the height of the system (above the papillary muscles). The following step is the leaflet capture, done by releasing the ventricular anchors. Once the valve is expanded, the leaflet capture is finalized. If the correct position is confirmed, the valve is deployed.

However, the femoral–transseptal approach is much more complicated, requiring an arterio-venous loop and an inflated balloon advanced from the left atrium to the LVOT to ensure that the wire is not caught in the mitral apparatus. The arterio-venous loop helps to position the valve.

The initial study, First in Human (FIH), showed encouraging results (25), and in 2015, two feasibility studies started in Europe and USA. Nevertheless, the recruitment stopped in 2017 due to company decision. The 1-month mortality was quite high (26.9%), with three procedure-related deaths and a technical success rate of 84.6% (22/26 patients) (13, 26).

#### Sapien M3—Edwards TMVR

Sapien M3 device is another transfemoral percutaneous mitral valve from Edwards. The valve is identical to aortic Sapien 3 29 mm, with the addition of an expandable polytetrafluoroethylene-covered nitinol “dock,” which encircles the chordae tendineae and native mitral valve leaflets, being the principal mechanism of anchoring (Table 2). After a transseptal puncture, a deflectable sheath is placed in the left atrium, and a steerable catheter is then advanced just under the posteromedial mitral commissure. The “dock” is a single component with three distinct sections that capture the chords and create an “artificial annulus” where the Sapien valve will be implanted. Moreover, the device presents a knitted (polyethylene terephthalate) cloth outside of the valve frame, which may avoid paravalvular leaks. The FIH study (27) was recently published, showing a technical success of 90% (9/10 patients) without any mortality at 30-day follow-up. The patients improved their NYHA class, and no re-admissions for HF were described.

#### Caisson TMVR System

The Caisson TMVR System, just as Sapien M3 and CardiaQ valves, is delivered *via* transfemoral approach. The valve design includes two separate components: a stent (anchor) and the valve (28). The anchor is a D-shaped, self-expanding nitinol structure, which is implanted at the level of the native mitral annulus, and it is the backbone for the bioprosthesis (Table 2). The ventricular part is engaged under the mitral valve annulus, and the atrial segment is anchored at the atrial surface of the mitral valve annulus. The valve is a trileaflet porcine valve percutaneously implanted using the transfemoral approach. The PRELUDE (NCT02768402) FIH study was finalized in 2018, and the preliminary results were positive (26), but the complete information regarding the follow-up is still being awaited. In addition, two studies [INTERLUDE (NCT03661398)—CE MARK trail and ENSEMBLE United States pivotal trial with the FDA protocol] should have started. In 2019, the company decided to stop the production of the valve.

#### HighLife TMVR System

The HighLife System uses the “valve in ring” concept where the ring is implanted *via* transfemoral (subannular position) approach, and the valve is placed inside the ring *via* transapical approach during the same procedure (29) (Table 2).

First, a guidewire is advanced through the femoral artery (18-Fr introducer) into the left ventricle and is looped around the native valve leaflets (guided by TEE). A “ring” is placed over the guidewire and it serves to anchor and avoids the displacement of the valve into the left ventricle. The valve is coupled with the ring at the level of a groove in the annular region. This way, the native leaflets are trapped between the subannular implant and the prosthetic valve (4). The ring is in a subannular position to prevent LVOT obstruction by pulling and fixing the anterior mitral leaflet instead of pushing it into the LVOT (30).

The available results include a cohort of 15 patients, with a technical success of 72.7% and procedure-related mortality of 18.2% (26). The trial is still active but not recruiting.

#### Fortis TMVR System

The Fortis TMVR System is a cloth-covered self-expanding nitinol frame with a trileaflet bovine valve whose anchoring system consists of two opposing paddles, which must be placed in the A2-P2 area (Table 2). Additionally, the device presents an atrial flange and is made of multiple nitinol struts (3, 31). The valve is implanted through a 42-Fr transapical access, without need for rapid pacing. Until 2015, 13 compassionate use cases were performed. The high cardiovascular mortality (38.5%) (3) and valve thrombosis made the company determined to halt the valve production temporarily.

#### Cardiovalve TMVR System

The Cardiovalve TMVR is a self-expandable valve, delivered through a 28-Fr introducer *via* transfemoral– transseptal approach using a multi-steerable catheter for coaxial implantation and without any atrioventricular loop (32). The valve design mimics the surgical Edwards Permimont Magna valve for the mitral valve, and it has similar characteristics: low ventricular profile with no atrial protruding, anchoring system and sealing elements, and three differently sized valves with diameters that range from 40 to 50 mm^2^ (Table 2). The results of the first five patients were recently presented, showing a technical success rate of 100%. Nevertheless, the 30-day mortality was 60% due to vascular complications (2). The AHEAD (European Feasibility Study of the Cardiovalve Transfemoral Mitral Valve System; NCT03339115) study is currently recruiting patients to evaluate the safety and the device performance of the Cardiovalve system.

#### Evoque TMVR System

The Evoque system is another mitral valve device delivered through transfemoral– transseptal access, designed by Edwards Lifesciences. It consists of a self-expanding nitinol frame and bovine pericardial leaflets. The ventricular outflow portion presents anchors used to engage the mitral leaflets and the subvalvular apparatus. The atrial inflow portion has a skirt to minimize paravalvular leaks. The delivery system allows the flexion to cross the interatrial septum and mitral valve, the depth control function ensures valve alignment, and the stabilizer stand controls the deployment. The initial experience was recently published, showing a technical success of 92.2% (13/14 patients) without any cardiovascular mortality at 30-day follow-up. The PVL was the main complication, requiring conversion to surgery in one case and percutaneous closure in other two cases. Moreover, there is also a concern regarding valve thrombogenicity since, in two of four patients with CT follow-up, hypoattenuated leaflet thickening with increased gradient was seen (33).

## Devices in the Early Phase of Development

There are few devices in developing or preclinical studies. Three of them have had at least one First in Human case: The NaviGate (NaviGate Cardiac Structures Inc.) valve (34), the MValve (MValve Ltd., Israel), and the AltaValve (4C Medical Technologies Inc.) (35). The first two are no longer used. The Navigate valve was abandoned for mitral use and is currently participating in feasibility studies for tricuspid regurgitation (36). The MValve is composed of a docking system (for anchoring), where a percutaneous valve is implanted (Lotus valve). Since this valve was no longer commercialized, the device has been put on hold.

The AltaValve has a unique design and consists of a self-expanding supra-annular device, with a 27-mm bovine tissue valve mounted into a nitinol frame of spherical shape (50 to 90 mm), partially covered by a fabric skirt (35). Being a supra-annular device with only atrial fixation, it may eliminate potential complications such as LVOT obstruction and embolization. However, anchor shape has two important drawbacks: the potential risk of thrombogenicity since there is more material in the atrium and the difficulty of accessing the left appendage if needed.

Other technologies such as the Cephea (Cephea Valve Technologies) system, AccuFit system (Sino Medical Science Technology, China), Saturn technology (HT Consultant, Switzerland), and MitrAssist Valve (MitrAssist Ltd., Israel) are still in preclinical studies.

### Devices for Native Calcified Mitral Valves (Valve in MAC—Vi-MAC)

Mitral annular calcification is a degenerative process, and its quantification has still not been validated. The presence of diffuse, almost circumferential heavy calcification of the mitral valve ring evaluated using CT was considered as severe MAC. Moreover, a total volume of 750 mm^3^ was also defined as severe MAC (18).

Mitral annular calcification may represent an intimidating surgical challenge during mitral valve surgery, and most of the patients with MAC are conservatively treated. Major bleeding, atrioventricular disruption, and ventricular rupture are just some of the fearsome complications. Moreover, patients with severe MAC are elderly and at a very high risk for surgery. Hypothetically, TMVR should be a less invasive procedure, but up to date, there are no specific devices designed for MAC. The aortic balloon–expandable valve may be used as off-label in MAC cases (37–39). The TMVR in MAC Global Registry is the largest study using Sapien valve (Edwards Lifesciences, Irvine, California) and included 116 patients from 51 centers. The acute technical success was 76.7%. The most frequent complication was LVOT obstruction with hemodynamic compromise in 11.2% of cases, which was an independent predictor of mortality (37). A total of 14.7% of patients needed a second valve, mainly due to the presence of residual mitral regurgitation. Moreover, a non-negligible number of patients needed re-intervention.

The mortality rate was 25% at 30-day and 53.7% at 1-year follow-up. The results should be interpreted with caution. These outcomes might have been related to patient selection (mean STS score of 15.3), and probably those patients were treated too late (>50% non-cardiovascular mortality). Almost all the survivors at 1-year follow-up experimented a clear symptoms improvement. The TMVR in MAC Global Registry also included a group of patients treated through the transarterial approach. Although much more invasive, this technique may have some advantages in cases that cannot be performed *via* transseptal or transapical approach. It permits to resect part of the anterior leaflet or septum if needed and provide better anchoring and alignment because pledged sutures can be placed. In experienced hands, the technique showed favorable results (40, 41).

Recently, Sorajja et al. (18) presented the first experience with the Tendyne valve in MAC patients, showing encouraging results. The acute technical success was 89%, with no cardiovascular mortality at 1-year follow-up. Both Tendyne and Intrepid valve will be used during the randomized studies SUMMIT trial (NCT03433274) and Apollo Trial (NCT03242642), respectively.

### Devices for Failed Prosthetic Ring and Band (Valve in Ring, MViR)

Mitral valve repair is the elected treatment for patients with degenerative severe mitral regurgitation. Although the initial results are excellent, at 20-year follow-up, 15% of them present moderate–severe mitral regurgitation. In the last few years, patients at a high risk for re-surgery and failed mitral annuloplasty underwent transcatheter mitral valve implantation using percutaneous aortic valves, with acceptable results (42–44). Nevertheless, the procedural success may differ depending on the type of mitral annuloplasty (bands of rings, complete or incomplete, rigid or semi-rigid). A few details that should be taken into consideration are as follows: (1) bands, due to their texture, may not give sufficient support for valve anchoring, (2) rigid rings may deform the THV and may also lead to paravalvular leakage, (3) ring size >32 mm is too large for the current THV, and (4) the risk of delayed embolization since the valve may “slipper” in a ring that cannot offer enough fasten or partial dehiscence of the ring due to the mechanical forces of the THV. In the MITRAL Trial (44), THV size selection was made based on the mitral annular area in the majority of cases. Technical success was obtained in 70% of patients, and the 30-day mortality was 6.8%. A second valve was needed in 20% of cases (in the early experience), and it was not associated with poor outcome. The TMVR registry (45) and a recent meta-analysis (46) showed a lower technical success of MViR compared with MVIV. The Sapien valve was the most common device used. However, anecdotic cases with Lotus valve, Direct Flow, or Melody were also described (46).

### Devices for Failed Bioprosthesis (Valve in Valve—MViV)

During the first 10 years after mitral valve replacement, up to 35% of patients may require a repeat operation (47). In the best scenario, the durability of the mitral bioprosthesis may reach 16.6 years (47), which means that many of those patients will need a second surgery by the age of 75–80 years. Comorbidities, clinical presentation, and advanced age can make the second surgery extremely risky. Back in 2009, Cheung et al. (48) described the first TMVIV implantation in humans. Nowadays, TMViV is more than an accepted option for those patients with a high risk for re-surgery in degenerated bioprosthesis. The SAPIEN 3 MViV registry, recently published (49), is the largest registry of failed mitral bioprosthesis treated with THV (1,529 cases treated with Sapien 3 Valve). The transseptal access was the main access site for THV implantation (86.7%), with no difference regarding the technical success results between the transseptal and the transapical routes (97.1 vs. 94.6%; *P* = 0.8). Although there were no differences in the in-hospital endpoints of stroke, mitral valve re-intervention, new pacemaker, peri-procedural MI, or major vascular complications, the transseptal access proved to be more advantageous in terms of cardiovascular death (1.8 vs. 4.4%; *P* = 0.03), median length of stay (2 vs. 6 days; *P* < 0.001), and discharge to home (82.5 vs. 59.1%; *P* < 0.001). At 1-year follow-up, the mortality was 16.7%, and transseptal access was an independent predictor of lower mortality compared to transapical access. Finally, the most important challenge during THV implantation remained LVOT obstruction.

The Mayval valve, of which the design is almost identical to the Sapien valve, could also be used for MViV. Although anecdotal cases were performed using this valve, there are no reports in the literature.

### Potential Complications During and After TMVR

Although TMVR is a less invasive procedure than conventional mitral surgery, it presents several complications (Table 3). Some of them are related to the learning curve (bleeding, thrombogenicity, *etc*.), and some are common with the surgical replacement.

**Table 3:** Follow-up after TMVR and complications.

	**Tendyne**	**Intrepid**	**TIARA**	**CardiaQ**	**Sapiens 3M**	**Caisson**	**Highlife**	**Fortis**	**Cardio valve**	**Evoque**
Number of patients	100	50	73*	26	10	11	15	13	5	14
Mean Follow-up, mo	13.7	7.04	1	1	1	9.9	12	24	12	1
Mortality (%)	26/100 (26)	11/50 (22)	8/71 (11.3)	7/26 (26.9)	0/10 (0)	2/11 (18.2)	4/15 (26.7)	7/13 (53.8)	3 (60)	1 (7.1)
Cardiovascular mortality	22/100 (22)	11/50 (22)	6/71 (8.5)	NA	0/10 (0)	NA	NA	5/13 (38.5)	3 (60)	0/14
NYHA III-IV	10/86 (11.6)	9/43 (20.9)	NA	NA	1/9 (11)	1/9 (11.1)	NA	1/8 (12.5)	1 (50)	2 (18.2)
Mean transmitral gradient mmHg	3.0 ± 1	4.1 ± 1.3	NA	NA	6	3.1	NA	3 ± 1	3.4 ± 1.7	5.8
Moderate-severe MR	0/100 (0)	0/42 (0)	0/9 (0)	NA	1/9 (11)	1/11 (9.1)	0/12 (0)	0/8 (0)	0/5 (0)	0/11 (0)
Stroke	3/100 (3)	3/50 (6)	NA	NA	NA	0/11 (0)	0/15 (0)	0/13 (0)	0/5 (0)	2/14 (14.2)
Myocardial infarction	4/100 (4)	0/50 (0)	NA	NA	0/10 (0)	0/11 (0)	0/15 (0)	0/13 (0)	0/5 (0)	0/14 (0)
HF hospitalization	31/100 (31)	12/50 (15.4)	NA	NA	0/10 (0)	1/11 (9.1)	NA	2/13 (15.4)	0/5 (0)	0/14 (0)
PM implantation	7/100 (7)	NA	NA	NA	NA	NA	NA	NA	NA	1 (7.1)
BARC 2, 3, or 5 bleeding	32/100 (32)	9/50 (18)	NA	NA	1/10 (10)	0/11 (0)	NA	2/13 (15.4)	2/5 (40)	3/14 (21.4)
Device hemolysis	3/100 (3)	0/50 (0)	NA	NA	NA	NA	NA	NA	0/5 (0)	0/14 (0)
Device embolization	0/100 (0)	0/50 (0)	2/73 (2.7)	NA	NA	NA	NA	0/13 (0)	0/5 (0)	0/14 (0)
Device thrombosis	6/100 (6)	0/50 (0)	NA	NA	NA	NA	NA	NA**	0/5 (0)	2/14 (14.2)
Endocarditis	2/100 (2)	2/50 (4)	NA	NA	NA	NA	NA	NA	NA	0/14 (0)
LVOT obstruction	1/100	0/50 (0)	0/73 (0)	NA	NA	0/11 (0)	1/15 (6.6)	0/13 (0)	0/5 (0)	1 (7.1)

*HF, heart failure; Mo, months; MR, mitral regurgitation; NYHA, PM: pacemaker*.

* *Patients from TIARA I, TIARA II and compassionate use cases*.

** *Fortis valve is not currently available. Valve thrombosis was often documented*.

#### Left Ventricular Outflow Tract Obstruction

The Neo-LVOT area is the area that remains after mitral surgery or TMVR and decreases after all these procedures (50, 51). LVOT obstruction is one of the most fearsome complications and is potentially deadly, with 62% of in-hospital mortality (19). Various factors may determine the LVOT obstruction: device protrusion into the left ventricle, aorto-mitral angle (an angle <120° in peak systole may predispose to LVOT obstruction post-TMVR), degree of septal hypertrophy, left ventricle size, anterior leaflet displacement after valve implantation, a long anterior mitral leaflet with redundant chordae, and the amount of calcification in MAC cases. The described rate of LVOT obstruction is 2.2% for MViV, 5% for MViR (45), and 39.7% in ViMAC, respectively (19, 52). Taking into consideration the lessons learned from these studies, the screening failure for TMVR is as high as 40% in the Intrepid Global Pilot Study (22) and 60% in a French registry (53).

Moreover, the assessment of LVOT obstruction risk is based on CT measurements. A neo-LVOT area <250 mm^2^ at end-systole (22) was considered as a contraindication for TMVR implantation and a neo-LVOT area <170 to 190 mm^2^ at mid- to late-systole predicted a high risk of LVOT obstruction for MViV, MViR, and Vi-MAC (54). Recently, Meduri et al. (51) showed that multiphase and specifically early systolic assessment of the neo-LVOT might better determine the risk of LVOT obstruction after TMVR compared with end-systolic measurements. Currently, only two cases of LVOT obstruction were described with the new THV (one case with Tendyne valve and another with Highlife Valve).

The specific design of the THV overcome in part to this potential complication: the Intrepid valve, due to its lower profile (height <18 mm), may be used even in “relatively contraindicated conditions” as prior prosthetic aortic valve replacement and with a smaller ventricular size; the Highlife valve, with its “valve in ring” design, allows to trap the native leaflets between the sub-annular implant and the prosthetic valve, which may prevent LVOT obstruction by pulling and fixing the anterior mitral leaflet instead of pushing it into the LVOT. The unique design of the AltaValve, with the only fixation at the atrial level, reduces to minimum the LVOT obstruction risk.

Moreover, different techniques were described to avoid or to treat LVOT obstruction during TMVR: alcohol septal ablation (37), radiofrequency septal ablation using SCORPION technique (55), laceration of the anterior mitral leaflet (LAMPOON technique) (56, 57), and balloon-assisted translocation of the anterior mitral leaflet (BATMAN technique) (58).

Alcohol septal ablation was performed as a bailout procedure in those patients with LVOT obstruction after TMVR implantation, with acceptable results (37). Nevertheless, it may cause conduction disturbance, and in some cases, it might not be feasible due to inadequate septal thickness. Ongoing studies try to prove the role of prophylactic alcohol septal ablation in those cases at a high risk for LVOT obstruction, taking into consideration that changes in the septum may delay between 4 and 6 weeks after ablation.

The SCORPION procedure is a novel septal ablation technique (55). Two ablation catheters are placed at the level of the septum in the right and the left ventricles, and multiple applications at 35 W are performed. Three patients underwent this procedure with important reduction of the ventricular mass, but with a rate of pacemaker of 100%.

The LAMPOON procedure emerges as a feasible technique to avoid LVOT obstruction during TMVR in those “contraindicated” cases. It is performed during TMVR implantation and consists of a controlled transcatheter laceration of the anterior mitral leaflet. Two guiding catheters are advanced using arterial femoral access and placed onto the left ventricle and left atrium, respectively. A stiff 0.014-in. guidewire (Astato XS 20, Asahi, Japan) is sheathed in an insulating polymer jacket (Piggyback Wire Convertor, Teleflex, North Carolina) and advanced from the LVOT to perforate through the center and the base of the anterior mitral leaflet using a short pulse of radiofrequency energy. Then, it is snared into the guiding catheter localized in the left atrium. The wire (electrified) is externalized, lacerating the AML by pulling on the two catheters. As a result, anterior mitral leaflet splays in diastole and coapts in systole. Khan *et al*. described the LAMPOON technique in 30 patients, with a laceration success of 100% (57).

The BATMAN technique mimics the surgical approach called “translocation of the anterior mitral leaflet with chordal preservation,” with a less invasive access (58). It has similar principles to the LAMPOON technique. Through transapical access, a pericardiocentesis needle is advanced, puncturing the anterior mitral leaflet (optimal puncture in the middle, at an equal distance between the tip and the base of the leaflet). Posteriorly, a 0.035-in. stiff wire is placed into the left superior pulmonary vein, and a 20-mm balloon is advanced and inflated, creating a hole in the anterior mitral leaflet. Through the same wire, the THV is then advanced and deployed, avoiding the displacement of the bulky anterior mitral leaflet into the LVOT. Up to date, the procedure is performed only under cardiopulmonary bypass and using transapical access.

Patients who underwent Mitraclip with persistent residual or recurrent mitral regurgitation are challenging cases. Usually, these patients are not eligible for conventional surgery, and a THV is contraindicated due to the presence of a clip. However, a new transcatheter electrosurgery technique was reported and may allow for the selective laceration of failed Mitraclip and subsequent placement of a dedicated THV (59). Three successful cases were described. The MitraClip anterior leaflet laceration (ELASTA-Clip) technique is performed using a wire, which surrounds the anterior leaflet. The wire is connected to a radiofrequency source and then into the left atrium. Following anterior leaflet laceration, the clip(s) remains selectively attached to the posterior leaflet, and THV is implanted.

#### Bleeding

Bleeding occurs in 10–40% of patients after TMVR (Table 3) due, in most cases, to the transapical approach, and it is correlated with high morbidity and mortality. The use of large-bore access sites (>30 Fr) and anticoagulation treatment may facilitate bleeding despite two purse-string sutures with felt pledgets at the access level. The Tendyne valve presents an epicardial pad, which helps to promote hemostasis and reduce the risk of access bleeding. The Intrepid procedure had a higher rate of bleeding, which may be explained in part by the intensive anticoagulation and antiplatelet therapy after TMVR.

#### Hemolysis and Paravalvular Leaks

Hemolysis is a less frequent complication and may occur after TMVR in the presence of paravalvular leak as a result of turbulent flow pattern and erythrocyte destruction. The presence of hemolysis was described in three Tendyne cases (1). The rest of the studies did not report the rate of hemolysis.

The incidence of paravalvular leaks and hemolysis may be higher in MViR and Vi-MAC because the THV does not have the same shape as the native valve/mitral annuloplasty and gaps may remain in between. The treatment can be percutaneous or surgical. There are several cases which were successfully treated with AVP devices. Surgery remains the last option since the patients are at a high risk.

#### Endocarditis

The endocarditis rate at 1-year follow-up was 4%, and it was reported in the Tendyne, Intrepid, and S3 MViV studies. Prophylaxis should be done as for regular bioprosthesis.

#### Trombogenicity

The experience with mitral bioprosthesis showed the need for oral anticoagulation after surgical mitral valve replacement for 3–6 months (60, 61). The rationale for anticoagulation after mitral valve replacement/implantation is to reduce the risk of thromboembolic events (stroke, myocardial infarction of valve thrombosis) until the valve is fully endothelialized. Moreover, the turbulent flow around the valve, the pre-existing pro-thrombotic conditions, and new atrial fibrillation may increase the risk of arterial embolism.

The antithrombotic treatment after TMVR is ambiguous since only three studies (1, 3, 16) reported it. Initially, single antiplatelet therapy with aspirin was recommended after Tendyne valve implantation (patients with no need for oral anticoagulation). Due to the relatively high rate of THV thrombosis (6%), the protocol was changed, and anticoagulation with INR between 2.5 and 3.5 during the first 3 months was required. Another two cases were described with the HighLife and Fortis valves, summing up to 7% for each valve. The Fortis THV program was stopped in 2015 because of issues related to device thrombosis (62).

Moreover, the rate of valve thrombosis in the MViV and MViR groups may reach 15.4% (62) (often in patients with single antiplatelet therapy), and recent experience with ViMAC showed 1.3% of THV thrombosis (62). Valve thrombosis is a serious complication, which may be silent or may give heart failure symptoms, and its treatment is anticoagulation. There are no strict recommendations regarding antithrombotic therapy after TMVR. It seems that anticoagulation treatment with VKA for at least 3 months is beneficial. In those cases which are at a very high risk for bleeding, single antiplatelet therapy may be an option, but strict clinical and imaging follow-ups should exclude the occurrence of THV thrombosis. Finally, stroke and myocardial infarction events were described in the Tendyne and Intrepid groups, and they were mainly related to the procedure.

#### Pacemaker Implantation

The need for pacemaker implantation after TAVI is 10–30%. Nevertheless, there is no data in the field of TMVR. Hypothetically, it should be lower since no predilatation is needed, and the valve is placed far from the septum. The Tendyne registry reported 7% of pacemaker implantation, while the other studies did not mention it.

#### Embolization, Migration, Malposition

This phenomenon is mainly related to the imperfect match between the THV and the mitral annulus, previous bioprosthesis, ring, or band. Moreover, there are several THVs with a distinct site of anchoring: at the level of the mitral valve involving the leaflets or not and at the level of the apex or the left atrium. In the native valve, the absence of calcification and the D-shape makes perfect anchoring difficult. The only THV registries which reported delayed migration were TIARA I and TIARA II, which together presented a rate of 2.7% (23). Late embolization was also observed in the MViR group, and it may be explained by the mechanical force and possible dehiscence of the ring or band.

## Conclusions

Currently, it was proven that the percutaneous transcatheter aortic valve replacement is feasible and comparable with surgical series, and the percutaneous transcatheter mitral valve replacement is just feasible for now. The development of mitral devices is a more complex process. The mismatch between mitral anatomy and prosthesis characteristics determine almost 60% of screening failure. From experience gained with fewer than 1,000 TMVR performed worldwide, we learned the following:

1. TMVR is an acceptable option for patients with mitral valve disease and who are at a high risk for surgery with a rate of technical success at >80%.
2. Mortality at 1-year follow-up is comparable with Mitraclip population, although it is high and mainly related to procedural complications.
3. The transapical approach permits “easy” valve deployment, with a higher risk of access bleeding.
4. During the first 3 months, anticoagulant treatment should be recommended to avoid potential complications such as valve thrombosis; nevertheless, the bleeding risk should be evaluated for each patient.
5. LVOT obstruction after valve implantation is the Achilles heel, and new techniques were described to overcome this fearsome complication.
6. The aortic THV for MViV, MViR, and Vi-MAC is feasible, with encouraging results at midterm follow-up.

Randomized trials comparing TMVR with traditional mitral surgery are ongoing, and their first results are expected at the end of 2021.

## Author Contributions

All authors listed have made a substantial, direct and intellectual contribution to the work, and approved it for publication.

## Conflict of Interest

MS is a consultant for Abbott Vascular. The remaining authors declare that the research was conducted in the absence of any commercial or financial relationships that could be construed as a potential conflict of interest.
